# Differences in Epidemiological and Molecular Characteristics of Nasal Colonization with *Staphylococcus aureus* (MSSA-MRSA) in Children from a University Hospital and Day Care Centers

**DOI:** 10.1371/journal.pone.0101417

**Published:** 2014-07-02

**Authors:** Erika A. Rodríguez, Margarita M. Correa, Sigifredo Ospina, Santiago L. Atehortúa, J. Natalia Jiménez

**Affiliations:** 1 Grupo de Microbiología Molecular, Escuela de Microbiología, Universidad de Antioquia, Medellín, Colombia; 2 Grupo de Investigación en Microbiología Básica y Aplicada - MICROBA, Escuela de Microbiología Universidad de Antioquia, Medellín, Colombia; 3 Hospital Universitario de San Vicente Fundación, Medellín, Colombia; Rockefeller University, United States of America

## Abstract

**Background:**

Clinical significance of Staphylococcus aureus colonization has been demonstrated in hospital settings; however, studies in the community have shown contrasting results regarding the relevance of colonization in infection by community-associated MRSA (CA-MRSA). In Colombia there are few studies on *S. aureus* colonization. The aim of this study was to determine the molecular and epidemiological characteristics of nasal colonization by *S. aureus* (MSSA-MRSA) in children from a university hospital and day care centers (DCCs) of Medellin, Colombia.

**Methods:**

An observational cross-sectional study was conducted in 400 children (200 in each setting), aged 0 months to 5 years, during 2011. Samples were collected from each nostril and epidemiological information was obtained from the parents. Genotypic analysis included *spa* typing, PFGE, MLST, SCC*mec* typing, detection of genes for virulence factors and *agr* groups.

**Results:**

Frequency of *S. aureus* colonization was 39.8% (*n* = 159) (hospital 44.5% and DCCs 35.0%) and by MRSA, 5.3% (*n* = 21) (hospital 7.0% and DCCs 3.5%). Most *S. aureus* colonized children were older than two years (*p* = 0.005), the majority of them boys (59.1%), shared a bedroom with a large number of people (*p* = 0.028), with history of β-Lactamase inhibitors usage (*p* = 0.020). MSSA strains presented the greatest genotypic diversity with 15 clonal complexes (CC). MRSA isolates presented 6 CC, most of them (47.6%) belonged to CC8-SCC*mec* IVc and were genetically related to previously reported infectious MRSA strains.

**Conclusion:**

Differences in epidemiological and molecular characteristics between populations may be useful for the understanding of *S. aureus* nasal colonization dynamics and for the design of strategies to prevent *S. aureus* infection and dissemination. The finding of colonizing MRSA with similar molecular characteristics of those causing infection demonstrates the dissemination capacity of *S. aureus* and the risk of infection among the child population.

## Introduction

*Staphyloccocus aureus* is one of the principal human pathogens, responsible for various types of important infections in the community and hospital settings [1]. This microorganism is characterized by its high capacity to adapt to antimicrobials by the acquisition of resistance mechanisms particularly against methicillin, further complicating the treatment of infections [1].

Besides its advantages as a pathogen and its capacity to develop resistance mechanisms, *S. aureus* presents a great ability to colonize humans, primarily their nose [2]. Colonization is an important factor in the pathogenesis and epidemiology of infections by *Staphyloccocus aureus* methicillin-sensitive (MSSA) and methicillin-resistant (MRSA) [2]. It is suggested that there is a greater risk for previously colonized individuals of developing infection or of invasive infection after colonization by MRSA [3]. Children are particularly susceptible to colonization by *S. aureus* with prevalences that vary from 7.6–53.8%, depending on the age group [4], [5]. Furthermore, they generally present a pattern of persistent colonization and may act as vectors disseminating *S. aureus* throughout the community and in healthcare institutions [6].

The importance of colonization has been defined in more detail in hospital environments, while its significance in the community is still controversial. It is suggested that colonization has little relevance to pathogenesis and infection by community-associated MRSA (CA-MRSA) [7]. However, an increase in nasal colonization has been implicated as the principal risk factor in the emergence of MRSA infections, especially in healthy children [8], [9]. In Colombia and particularly in Medellin, few studies have been carried out on *S. aureus* colonization in children or to describe the molecular characteristics of the colonizing strains. Considering that the epidemiology of *S. aureus* depends on the particular conditions of each population, the objective of this study was to determine the molecular and epidemiological characteristics of nasal colonization by *S. aureus* (MSSA-MRSA) in children from a university hospital and day care centers (DCCs) in the city of Medellin, Colombia.

## Materials and Methods

### Study Population

An observational cross-sectional study was conducted in children aged 0 months to 5 years admitted to the Pediatric Department of Hospital Universitario de San Vicente Fundación (HUSVF) and from eight DCCs of Medellin, the second largest city of Colombia, during 2011. The research and informed consent protocols for this study were approved by the Bioethics Committee for Human Research of the University Research Center, Universidad de Antioquia (CBEIH-SIU-UdeA) (approval No 10-041-277), as well as by the Research Ethics' Committee of HUSVF. Written informed consent to participate in the study was obtained from the children parents or guardians prior to sample collection. Hospital Universitario de San Vicente Fundación is a fourth-level care center with 648 beds, and its Pediatric Department has 186 beds. The children included in the study were randomly selected from the different services of the Pediatric Department and included, general hospitalization, nursing, oncology and nephrology. According to data from the HUSVF-Clinical Microbiology Service, during 2010 the prevalence of MRSA in all types of infections was 31.8%. The eight DCCs (A–H), are located in neighborhoods of low socio-economic status and belong to the “Buen comienzo” (Good Start) program sponsored by the municipality government. The number of children attending the DCCs varied, A (*n* = 100), B (*n* = 55), C (*n* = 90), D (*n* = 75), E (*n* = 150), F (*n* = 75), G (*n* = 100) and H (*n* = 60).

Sample size was estimated based on an expected rate of 0.5 *S. aureus* positive children, for a total sample of 400 children (200 from each setting, hospital and DCCs). Children with more than 48 h of hospitalization or more than 6 months of attendance to the DCCs were included in the study. Children taken antibiotics during the previous seven days to sampling were excluded.

### Clinical and epidemiological data

Epidemiological information was obtained from the medical records and parents or guardians for each child. Information included demographic aspects, medical history, antimicrobial usage, history of previous hospitalization, comorbidities, number of family members, smokers in the household and other possible factors linked to colonization.

### Collection of nasal swabs and microbiological procedures

Samples from each nostril were collected using sterile cotton swabs with sterile 0.9% saline solution, rotated two or three times in the vestibule of both anterior nares and immediately placed in Amies transport medium with charcoal, conserved at 4–8°C and transported to the microbiology laboratory within 4 h of collection [10]. In the lab, samples were immediately inoculated onto mannitol-salt agar, incubated at 37°C, for 24–48 h. Colonies with mannitol-salt fermentation and morphology suggestive of *Staphylococcus* were subcultured onto blood agar plates. Gram staining, catalase and rabbit plasma coagulase tests were performed [11].

### Molecular typing

Previous to molecular typing, confirmation of *S. aureus* and methicillin resistance was performed by amplification of species-specific *nuc* and *fem*A genes, and of *mec*A gene encoding resistance to methicillin, as previously described [12], [13].

*Spa* typing was performed on all MRSA and on fifty percent of MSSA strains randomly selected (69 isolates, 39 from hospital and 30 from DCCs) [14]. Amplification products were sequenced and *spa* types were determined using Ridom Staphtype software (version 1.4; Ridom, GmbH, Wurzburg, Germany [http://spa.ridom.of/inofx.shtml]).

MLST was performed on a subset of 10 isolates representing the more frequent *spa* types (11% of all *spa* typed isolates (*n* = 90) [15]. Allele numbers and sequence types (ST) were assigned using the database maintained at http://saureus.mlst.net/, while CC were inferred using eBURST analysis [16]. CC for the strains not processed by MLST were inferred by *spa* repeat pattern analysis [17], [18] or by referring to the Ridom Spa Server website.

SCC*mec* types and subtypes for MRSA isolates were determined using a set of multiplex PCR reactions [19], [20].

Pulsed-field gel electrophoresis (PFGE) was performed on a representative subset of 64 colonizing *S. aureus* isolates, corresponding to all MRSA (*n* = 21) and 43 MSSA isolates randomly selected, representative of both settings and in a similar number of the resistant strains (22 from the hospital and 21 from DCCs) [21]. Digestion was carried out with *Sma*I enzyme. DNA fragment patterns were normalized using *S. aureus* strain NCTC 8325. Band assignments were manually adjusted after automatic band detection and only bands ranging from 36 kb to 600 kb were included in the analysis. Cluster analysis was performed using the Dice coefficient in BioNumerics software version 6.0 (Applied Maths, Sint-Martens-Latem, Belgium). Dendrograms were generated by the unweighted pair group method using average linkages (UPGMA), with 1% tolerance and 0.5% optimization settings. Similarity cutoffs of 80% and 95% were used to define types and subtypes, respectively [21]. Representatives of the most common infectious MRSA clones described in Colombia [22] and clone USA300-0114 CA-MRSA were used as reference strains.

### Detection of virulence factors and *agr* genes

All isolates were screened for the genes encoding staphylococcal enterotoxins (*sea, seb, sec, sed, see*), toxic shock syndrome toxin 1 (*tst*) and exfoliative toxins A and B (*eta*, *etb*), using the protocols and primers described by Mehrotra et al. [13] The identity of the *luk*S/F-PV genes enconding Panton-Valentine Leucocidine (PVL) was performed as previously reported [23]. Accessory gene regulator (*agr*) typing was amplified by Multiplex PCR to determine four types of *agr* [24]. The *arc*A gene coding for the arginine catabolic mobile element (ACME) was detected by PCR [23], [25].

### Statistical analyses

Comparisons of clinical, epidemiological and molecular characteristics were carried out between *S. aureus* colonized and non-colonized, and MSSA- and MRSA- colonized children. Categorical variables were compared using the Chi-square test or Fisher's exact test and Mann–Whitney U test for continuous variables. Values *p*≤0.05 were considered to be statistically significant. Multiple binomial regression analysis was applied to explore risk factors associated with *S. aureus* colonization in the overall population. Initially, a bivariate analysis was performed to estimate the prevalence ratios (PR) and the 95% confidence interval (CI). Variables that had a *p*-value <0.25 or that were epidemiologically important were included in the multivariate model, such as, institution, age, history of β-Lactamase inhibitors and passive smoking. Multiple binomial regression analysis of risk factors associated with colonization by MRSA was not performed due to lack of power as there were very few observations. Statistical analyses were carried out using the software package SPSS v20.0 (SPSS Inc., Chicago, USA) and Stata 11 (StataCorp, College Station, TX, USA).

## Results and Discussion

In Colombia few studies have been conducted to evaluate nasal carriage of S. aureus, and most of them have been performed in hospital settings evaluating healthcare personnel, and only recently, colonization in the pediatric population has been evaluated [26], [27]. This is the first study in Colombia, simultaneously characterizing, epidemiologically and molecularly, nasal colonization by *S. aureus* in two different pediatric populations, from the hospital and the community. The information provided is useful for the understanding of *S. aureus* nasal colonization dynamics in these populations and for the design of strategies to prevent *S. aureus* infection and dissemination.

### Frequency of nasal colonization by *Staphylococcus aureus*

The frequency of nasal colonization by *S. aureus* among the 400 children was 39.8% (*n* = 159) and of MRSA, 5.3% (*n* = 21). This findings are similar to previous reports for other countries of colonization frequencies among pediatric population of different ages, that vary between 7.6–53.8% [4], [5], and for MRSA between 0.3%–13.2% [28], [29]. Colonization at the hospital was 44.5% (*n* = 89), while in DCCs was 35.0% (*n* = 70) (*p* = 0.0659). Notoriously, a higher MRSA colonization frequency was observed in hospitalized children compared to DCCs, 7.0% (*n* = 14) vs. 3.5% (*n* = 7), although, this difference was no significant (*p* = 0.1786). These findings agree with other studies that have reported an increased frequency of MRSA colonization in healthy children [8], [9], and also indicate that DCCs are reservoirs that favor MRSA transmission in the community [30].

In previous studies in Latin America including children from DCCs of similar age to the ones in this study, the frequency of MRSA colonization varies, for example in Mexico, 0.93% [31], Brazil, 1.2% [6], Cuba, 2.2% [32] and Argentina 4.4% [33]. In the present study, MRSA colonization in DCCs children (3.5%), is similar to the frequencies detected in these countries, but lower than previously reported in other Colombian cities, e.g. 4.8% in Cartagena [26] and 12.6% in Montería [27]. Variations in colonization frequencies have been attributed to differences in socio-demographic characteristics [34]. Children included in this study belonged to low socioeconomic status neighborhoods, and for the studies in other Colombian cities only the one reporting higher frequencies (12.6%) [27], indicated that the children were from low socioeconomic neighborhoods. Although, the economic factor may influence colonization frequencies, other factors may be involved, such as the geographic background [34] and the number of anatomical sites sampled, thus, detection of MRSA carriers can be enhanced by taking samples from other body parts such as throat, skin, perineum, armpits and rectum [35]. In contrast to this work, in the Monteria study samples were taken from throat and nostrils, which in addition to the socioeconomic background, may have increased colonization frequencies.

### Epidemiological characteristics of the children colonized by *Staphylococcus aureus*

The epidemiological characteristics of children colonized by *S. aureus* in the overall population and by institution are described in Table 1. In general, children colonized by *S. aureus* were older than two years (*p* = 0.005), and the majority were boys (59.1%). In both populations, colonized children were more likely to share a bedroom with a large number of people (*p* = 0.028) and presented antecedents of β-Lactamase inhibitors usage (*p* = 0.020 for the general population, *p* = 0.038 for the hospital). Other characteristics such as sharing a towel (*p* = 0.011) were more frequently observed among the hospital children. Noteworthy, non-colonized, hospitalized children more likely used nasal sprays or washes (*p* = 0.038), or were vaccinated against *Streptococcus pneumoniae* (46.9%), as compared to colonized children (*p* = 0.023).

**Table 1:** Epidemiological characteristics of *S. aureus*-colonized children in the overall population and by institution (hospital - day care centers).

Characteristics				Hospital (n = 200)			DDCs (n = 200)		
	No. of *S. aureus*-colonized children (%)			No. of *S. aureus*-colonized children (%)			No. of *S. aureus*-colonized children (%)		
	Yes 159 (39.8)	No 241 (60.2)	*P^a^*	Yes 89 (44.5)	No 111(55.5)	*P^a^*	Yes 70 (35)	No 130 (65)	*P^a^*
Age:									
Median	3	2	**0.027^d^**	1	1	**0.006^d^**	3	2	0.207^d^
Range	(1–4)	(1–3)		(0–4)	(0–3)		(2–4)	(2–4)	
Age (years):									
>2	86 (54.1)	96 (39.8)	**0.005**	35 (39.3)	21 (18.9)	**0.001**	51 (72.9)	75 (57.7)	**0.034**
≤2	73 (45.9)	145 (60.2)		54 (60.7)	90 (81.1)		19 (27.1)	55 (42.3)	
Gender:									
Male	94 (59.1)	123 (51)	0.112	56 (62.9)	57 (51.4)	0.101	38 (54.3)	66 (50.8)	0.635
Female	65 (40.9)	118 (49)		33 (37.1)	54 (48.6)		32 (45.7)	64 (49.2)	
History of *S. aureus* in the previous year^ e^	-	-	-	0	2 (1,8)	0.503^b^	-	-	.
History in the previous year of:									
Hospitalization	74 (46.5)	103 (42.7)	0.454	63 (70.8)	82 (73.9)	0.627	11 (15.7)	21 (16.2)	0.936
Surgery	27 (17)	38 (15.8)	0.747	22 (24.7)	28 (25.2)	0.935	5 (7.1)	10 (7.7)	0.888
Dialysis	1 (0.6)	0 (0)	0.397^b^	0	0	_	1 (1.4)	0	0.350^b^
Stay in ICU	25 (15.7)	36 (14.9)	0.831	24 (27)	34 (30.6)	0.570	1 (1.4)	2 (1.5)	0.99^b^
Antimicrobial use in the previous 6 months:	78 (49.1)	114 (47.9)	0.821	47(52.8)	60 (54.1)	0.861	31 (44.3)	54 (42.5)	0.811
Penicillin	51 (65.4)	83 (72.8)	0.271	21 (44.7)	37 (61.7)	0.080	30 (96.8)	46 (85.2)	0.146^b^
Macrolides	7 (9)	17 (14.9)	0.222	6 (12.8)	14 (23.3)	0.164	1 (3.2)	3 (5.6)	0.99^b^
β-Lactamase inhibitors	16 (20.5)	10 (8.8)	**0.020**	16 (34)	10 (16.7)	**0.038**	0	0	-
Co-morbidities:	78 (49.1)	120 (49.8)	0.885	54 (60.7)	77 (69.4)	0.199	24 (34.3)	43 (33.1)	0.863
Diabetes Mellitus	1 (0.6)	2 (0.8)	0.99^b^	1 (1.1)	2 (1.8)	0.99^b^	0	0	_
Atopy	46 (28.9)	80 (33.2)	0.369	25 (28.1)	39 (35.1)	0.288	21 (30)	41 (31.5)	0.822
Neoplasia	4 (2.5)	6 (2.5)	0.99^b^	4 (4.5)	6 (5.4)	0.99^b^	0	0	_
Immunosu- pression	12 (7.5)	15 (6.2)	0.606	12 (13.5)	14 (12.6)	0.856	0	1 (0.8)	0.99^b^
Chronic renal disease	0 (0)	2 (0.8)	0.520^b^	0	2 (1.8)	0.504	0	0	_
Cardiovascular disease	6 (3.8)	6 (2.5)	0.553^b^	6 (6.7)	6 (5.4)	0.693	0	0	_
Chronic lung disease	4 (2.5)	5(2.1)	0.745^b^	4 (4.5)	5 (4.5)	0.99^b^	0	0	_
Malnutrition	13 (8.2)	11 (4.6)	0.137	11 (12.4)	11 (9.9)	0.582	2 (2.9)	0	0.121^b^
Pneumococcal conjugate vaccination	47 (33.1)	90 (40.7)	0.144	22 (29.7)	45 (46.9)	**0.023**	25 (36.8)	45 (36)	0.916
Influenza vaccination	130 (84.4)	204 (88.3)	0.269	70 (82.4)	89 (85.6)	0.546	60 (87)	115(90.6)	0.437
Family and personal history of SSTI in the previous year	62 (39)	80 (33.2)	0.236	36 (40.4)	43 (38.7)	0.806	26 (37.1)	37 (28.5)	0.207
Hospitalization of a family member within the previous 3 months	31 (19.6)	31 (12.9)	0.071	21 (23.6)	18 (16.4)	0.201	10 (14.5)	13 (10)	0.345
Contact with healthcare workers	18 (11.4)	40 (16.6)	0.149	13 (14.6)	24 (21.6)	0.204	5 (7.2)	16 (12.3)	0,269
Type of housing:									
Lodging	2 (1.3)	6 (2.5)	0.341^c^	2 (2.2)	6 (5.4)	0.597^c^	0	0	0.96^c^
Apartment	33 (20.8)	55 (22.8)		20 (22.5)	22 (19.8)		13 (18.6)	33 (25.4)	
House	113(71.1)	171 (71)		62 (69.7)	77 (69.4)		51 (72.9)	94 (72.3)	
Tenement house	11 (6.9)	8 (3.3)		5 (5.6)	5 (4.5)		6 (8.6)	3 (2.3)	
No. of occupants in the dwelling:									
Median	5	5	0.531^d^	5	5	0.623^d^	5	5	0.209^d^
Range	(4–7)	(4–6)		(4–7)	(4–7)		(4–7)	(4–6)	
No. of occupants in the bedroom:									
Median	3	3	**0.028^d^**	3	3	0.151^d^	3	2	0.188^d^
Range	(2–4)	(2–3)		(2–4)	(2–3)		(2–3)	(2–3)	
No. of minors sharing the dwelling:									
No. of children younger than 10:									
Median	3	3	0.137^d^	1	1	0.415^d^	3	3	0.188^d^
Range	(2–4)	(2–3)		(1–2)	(1–2)		(1–2)	(1–2)	
No. of minors between 11–18:									
Median	1	1	0.431^d^	1	1	0.485^d^	3	3	0.678^d^
Range	(1–2)	(1–2)		(1–1)	(1–2)		(1–2)	(1–2)	
Shared personal items:	63 (39.9)	99 (41.1)	0.810	26 (29.2)	1 (0.9)	0. 627	37 (53.6)	70 (53.8)	0. 976
Shared soap	59 (93.7)	96 (97)	0.432^b^	24 (92.3)	28 (96.6)	0.598^b^	35 (94.6)	68 (97.1)	0.608^b^
Shared towels	30 (47.6)	40 (40.4)	0.366	16 (61.5)	8 (27.6)	**0.011**	14 (37.8)	32 (45.7)	0.434
Mother's school grade:									
Elementary	62 (39)	80 (33.2)	0.178^c^	35 (39.3)	47 (42.3)	0.262^c^	27 (38.6)	33 (25.4)	0.130^c^
High school	69 (43.4)	102 (42.3)		39 (43.8)	35 (31.5)		30 (42.9)	67 (51.5)	
Higher education	9 (5.7)	31 (12.9)		4 (4.5)	12 (10.8)		5 (7.1)	19 (14.6)	
Illiterate	17 (10.7)	25 (10.4)		9 (10.1)	15 (13.5)		8 (11.4)	10 (7.7)	
No data reported	2 (1.3)	3 (1.2)		2 (2.2)	2 (1.8)		0	1 (0.8)	
Using nasal spray or nasal wash	47 (32.4)	70 (34.8)	0.640	37 (48.1)	60 (63.8)	**0.038**	10 (14.7)	10 (9.3)	0.277
Passive smoking	64(40.5)	82 (34)	0.189	38 (42.7)	37 (33.3)	0.174	26 (37.7)	45 (34.6)	0.667
Household pets:	59 (37.3)	78 (32.4)	0.306	32 (36)	42 (37.8)	0.784	27 (39.1)	36 (27.7)	0.099
Dog	44 (74.6)	53 (67.9)	0.398	25 (78.1)	28 (66.7)	0.279	19 (70.4)	25 (69.4)	0.937
Cat	16 (27.1)	17 (21.8)	0.471	10 (31.2)	10 (23.8)	0.475	6 (22.2)	7 (19.4)	0.787
Rabbit	2 (3.4)	4 (5.1)	0.699^b^	0	2 (4.8)	0.502^b^	2 (7.4)	2 (5.6)	0.99^b^
Birds	17(28.8)	25 (32.1)	0.684	13 (40.6)	20 (47.6)	0.549	4 (14.8)	5 (13.9)	0.99^b^
Other pets	4 (6.8)	9 (11.5)	0.347	3 (9.4)	6 (14.3)	0.723^b^	1(3.7)	3 (8.3)	0.629^b^

Significant differences (*p*<0.05) are shown in bold. Chi-square test^a^, Fisher's exact test^b^, likelihood ratio test^c^, Mann–Whitney U tests^d^. Variable evaluated only in hospitalized children^e^.

Multivariate analysis indicated that the variables that remained associated with an increased risk for *S. aureus* in the child population were, ages over two years (RP, 1.712; 95% CI, 1.384 to 2.118; *p* = 0.001), β-Lactamase inhibitors usage in the previous 6 months (RP, 1.655; 95% CI, 1.348 to 2.032; *p* = 0.001), exposure to cigarette smoke (RP, 1.361; 95% CI, 1.106 to 1.674; *p* = 0.004) and hospitalization (RP, 1.458; 95% CI, 1.133 to 1.876; *p* = 0.003) (Table 2). An additional factor that has often been correlated to *S. aureus* colonization frequencies is age. It has been reported that colonization decreases considerably during the first year of life, from 53,8% in the first month to 11,9% at 14 months [36]. In contrast, a gradual increase in colonization frequency is observed from two to five years of age [37], as observed in the present study. In addition, colonized children were exposed to cigarette smoke, which agrees with previous reports of an association of passive smoking with an increased risk of colonization in children [38]. Other epidemiological characteristics that have been correlated with *S. aureus* colonization and specifically MRSA are antibiotic usage in the previous 6 months and hospitalization [30]. Similarly, in this study, β-Lactamase inhibitors usage in the previous 6 months and hospitalization were also significant factors influencing colonization in the children population. These also constitute risk factors for *S. aureus* infection [3], [39], [40] and reinforces the importance of preventing *S. aureus* transmission in hospitalized children [41].

**Table 2:** Bivariate and multivariate analyses of risk factors associated with *S. aureus*- colonized children in the overall population.

Variable	Crude PR (95% confidence interval)	*P*	Adjusted PR (95% confidence interval)	*P*
Hospitalization	1.271 (0.995–1.623)	0.0659	1.458 (1.133–1.876)	0.003
Age (years) >2	1.411 (1.107–1.797)	0.0070	1.712 (1.384–2.118)	0.001
β-Lactamase inhibitors usage in the previous 6 months	1.647 (1.147–2.366)	0.0340	1.655 (1.348–2.032)	0.001
Passive smoking	1.179 (0.924–1.505)	0.2270	1.361 (1.106–1.674)	0.004

### Epidemiological characteristics of the children colonized by MRSA

Comparison of the epidemiological characteristics between children colonized by MRSA and by MSSA (Table 3), demonstrated that MRSA was more common among boys (*p* = 0.008). In the general population variables such as prior history of hospitalization (*p* = 0.047) and immunosuppression (*p* = 0.055) were related to colonization by MRSA. Most MRSA carriers had antecedents of surgery (*p* = 0.055 general population and *p* = 0.037 in hospital) and of sharing personal items (*p* = 0.027), especially among hospitalized children. In addition, in hospitalized, MRSA-colonized children antecedents of antibiotic usage (*p* = 0.007) and contact with healthcare workers (*p* = 0.029) were significant; in contrast, for children at DCCs, previous exposure to cigarette smoke (*p* = 0.010) and sharing a dwelling with children younger than 10 years (*p* = 0.0493) were the most frequent characteristics detected. These findings coincide with previous reports that show that colonization by MRSA is associated to characteristics such as male gender [11], previous history of hospitalization and surgery [42], previous contact with healthcare workers [8], [11], sharing personal objects [38] overcrowding conditions and high physical contact among children [37].

**Table 3:** Epidemiological characteristics of children colonized by MRSA and MSSA.

Characteristics	Total Population (*n* = 159) No. %			Hospital (n = 89) No. %			Day care centers (n = 70) No. %		
	MRSA positive No. (%) 21 (13.2)	MSSA positive No. (%) 138 (86.8)	*p^a^*	MRSA positive No. (%) 14 (15.7)	MSSA positive No. (%) 75 (84.3)	*p^a^*	MRSA positive No. (%) 7 (10)	MSSA positive No. (%) 63 (90)	*p^a^*
Gender									
Male	18 (85.7)	76 (55.1)	**0.008**	12 (85.7)	44 (58.7)	**0.054**	6 (85.7)	32 (50.8)	0.116^b^
Female	3 (14.6)	62 (44.9)		2 (14.3)	31(41.3)		1 (14.3)	31 (49.2)	
History in the previous year of:									
Hospitalization	14 (66.7)	60 (43.5)	**0.047**	13 (92.9)	50 (66.7)	**0.058^b^**	1 (14.3)	10 (15.9)	0.99^b^
Surgery	7 (33.3)	20 (14.5)	**0.055^b^**	7 (50)	15 (20)	**0.037^b^**	0	5 (7.9)	0.99^b^
Antimicrobial use in the previous 6 months:	13 (61.9)	65 (47.1)	0.206	12 (85.7)	35 (46.7)	**0.007**	1 (14.3)	30 (47.6)	0.123^b^
Co-morbidities:									
Immunosu-ppression	4 (19)	8 (5.8)	**0.055^b^**	4 (28.6)	8 (10.7)	0.091^b^	0	0	-
Malnutrition	4 (19)	9 (6.5)	0.073^b^	4 (28.6)	7 (9.3)	0.067^b^	0	2 (3.2)	0.99^b^
Contact with healthcare workers	5 (23.8)	13 (9.5)	0.068^b^	5 (35.7)	8 (10.7)	**0.029^b^**	0	5 (8.1)	0.99^ b^
No. of occupants in the bedroom:									
Median	3	3	0.056^c^	3	3	0.390^c^	4	3	0.072^c^
Range	(3–4)	(2–3)		(2–4)	(3–4)		(2–6)	(2–3)	
No. of children younger than 10 who share the dwelling:									
Median	2	1	0.085^c^	2	1	0.507^ c^	2	1	**0.0493^c^**
Range	(1–4)	(1–2)		(1–2)	(1–2)		(2–4)	(1–2)	
Shared personal items	13 (61.9)	50 (36.5)	**0.027^b^**	8 (57.1)	18 (24)	**0.022^b^**	5 (71.4)	32 (51.6)	0.437^b^
Passive smoking	11(52.4)	53 (38.7)	0.234	5 (35.7)	33 (44)	0.565	6 (85.7)	20 (32.3)	**0.010^b^**

Significant differences (*p*<0.05) are shown in bold. Chi-square test^a^, Fisher's exact test^b^, Mann–Whitney U tests^c^.

### Molecular characteristics of colonizing *Staphylococcus aureus* strains

**Figure 1:**
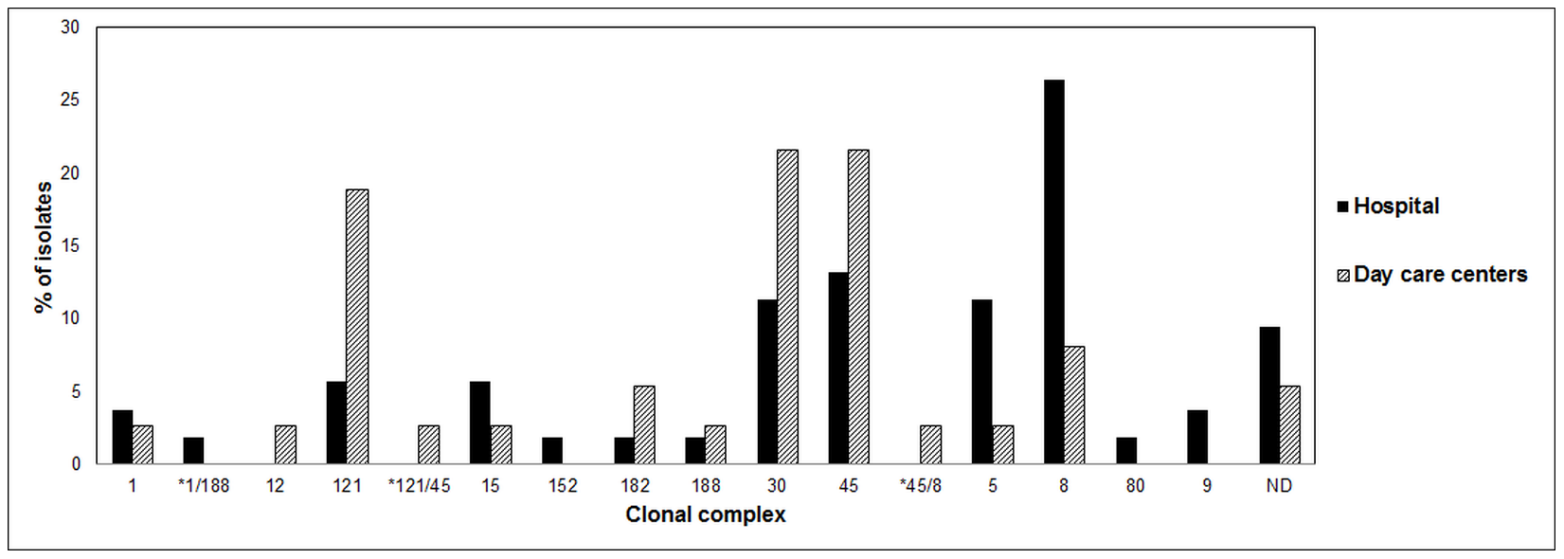
Clonal diversity of *Staphylococcus aureus* isolates among hospital and day care centers. The percent of isolates (Y-axis) is plotted against clonal complex type (X-axis). Abbreviations: ND: not determined. *Colonization with multiple *Staphylococcus aureus* strains with different clonal complexes.

Results of the molecular characterization of *S. aureus* colonizing pediatric population constitute one of the most relevant aspects of this work. Genotypes of *S. aureus* from hospitalized and DDCs populations revealed the presence of *S. aureus* strains with different molecular characteristics circulating in both settings; one of the main differences being their frequency of presentation (Fig. 1). Thus, isolates belonging to clonal complex CC8, CC30, CC45 and CC5 were most frequent in the hospital population and CC30, CC45 and CC121, in children of DCCs. The presence of CC30 and CC45 in both populations agrees with previous reports that indicate that these clonal complexes are among the most prevalent and successful colonizing strains [43], [44]. Further, isolates belonging to these clonal complexes have also been reported causing infection [45].

Among MRSA isolates (Table 4), six clonal complexes were determined, with predominance of CC8 (47.6%), CC45 (14.3%) and CC121 (9.5%). Furthermore, eleven *spa* types were detected, the most common were t024 (14.3%), t1610 (9.5%), t645 (9.5%), t1635 (9.5%) and t008 (9.5%). SCC*mec* typing showed that 33.3% (*n* = 7) of the MRSA strains carried SCC*mec* IVc, 23.8% (*n* = 5) SCC*mec* IVa and 42.9% (*n* = 9) were not typifiable.

**Table 4:** Molecular characteristics of MRSA isolates.

Clonal complex^a^	*spa* type^a^	SCC*mec*	*LukS/F-PV*	*arc*A	No (%)^b^	Population (No.)
1	t922	NT	-	-	1 (4.8)	D (1)
**121**	**t645**	NT	-	-	**2 (9.5)**	D (2)
182	t7862	NT	+	-	1 (4.8)	H (1)
**45**	New (X1-K1-A1-New-X2-B1-K1-B3-B3-A1-M1-B3)	IVa	+	−	1 (4.8)	H (1)
	t050	NT	+	−	1 (4.8)	H (1)
	t065	NT	+	−	1 (4.8)	D (1)
45/8	t065/t1635^c^	NT	+	−	1 (4.8)	D (1)
5	t002	IVa	−	−	1 (4.8)	H (1)
**8**	**t008**	IVc	+	−	**1 (4.8)**	H (2)
		NT	+	−	**1 (4.8)**	
	**t024**	IVc	+	−	**3 (14.3)**	H (3)
	**t1610**	IVc	+	−	**2 (9.5)**	H (1) D (1)
	**t1635**	IVa	+	−/+	**2 (9.5)**	H (2)
	t3308/t008^c^	IVc	+	−	1 (4.8)	H (1)
ND	New (Z1-B1-M1-E2-L1-J1-N1-N1-Q1-Q1)	NT	−	−	1 (4.8)	D (1)
	New (NEW-F1-G1-F1-M1-W2-W5)	IVa	+	−	1 (4.8)	H (1)

a The most prevalent clonal complexes and *spa* types are shown in boldface, defined by Ridom or e- genomics.

b Number and percentage of isolates with a specific clonal complex (CC), *spa* type and SCC*mec* type combination.

c Colonization with multiple *Staphylococcus aureus* strains with different *spa* type. NT: non-typeable, H: hospital, D: Day care centers.

**Figure 2:**
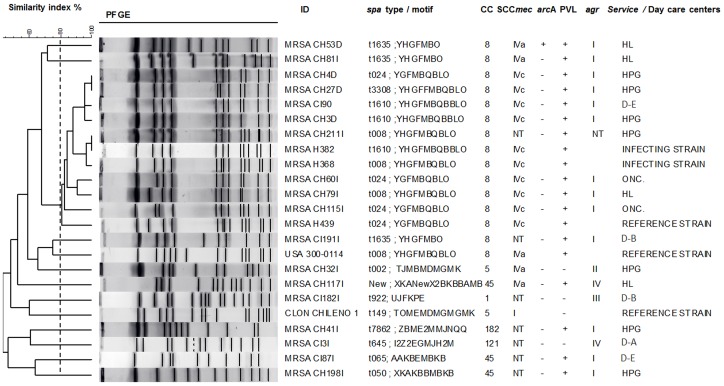
Genetic relatedness among MRSA isolates. UPGMA dendrogram showing genetic relatedness among MRSA isolates as determined by PFGE with *Sma*I. The broken line corresponds to the cutoff level (80%) used to define related PFGE clones. Note that CC8 MRSA isolates form a separate cluster by PFGE and were genetically related to infectious MRSA strains previously reported in the city.

PFGE analysis of all MRSA isolates showed a cluster of eight closely related isolates (coefficient of similarity 80–85%) (Fig. 2) that belonged to CC8, represented *spa* types t024, t008 and t1610 and SCC*mec* IVc, carried the PVL genes *lukS/F-PV* and *agrI*. Seven of these strains came from general hospitalization and oncology services, and one from a DCC (CC8-SCC*mec* IVc-t1610). Notoriously, these colonizing isolates were also closely related (coefficient of similarity 80–85%) to infectious isolates form a previous study, included in the PFGE assay as reference strains. Those strains represented the *spa* types t1610, t024 and t008, that were previously predominant in the hospitals, included the one evaluated here [22], [46]. These findings suggest the circulation and acquisition of these MRSA strains in the hospital environment. In addition, the finding of an infectious genotype colonizing children in a DCC, albeit at a low frequency, demonstrates its circulation among the general public; an issue of importance in public health given the pathogenic capacity and ability to disseminate of these type of strains. In the DCC population, a few MRSA strains belonging to CC1 and CC45 were detected. This is similar to results of the previous study on infectious MRSA that found a small proportion of these genotypes [22], [46]. Interestingly, two MRSA isolates belonging to CC121, not previously reported in Medellin were detected in DCCs. Notoriously, CC121 is uncommon among resistant isolates [45]. Finally, MRSA genotypes found in both populations differed from those detected at highest frequencies in colonized children of countries such as Brazil (CC8-ST239-SCC*mec* IVc-III) [6] and Argentina (CC5-ST5-SCC*mec* IV) [33]. Data that further evidences changes in the epidemiology of colonizing MRSA among different countries.

**Figure 3:**
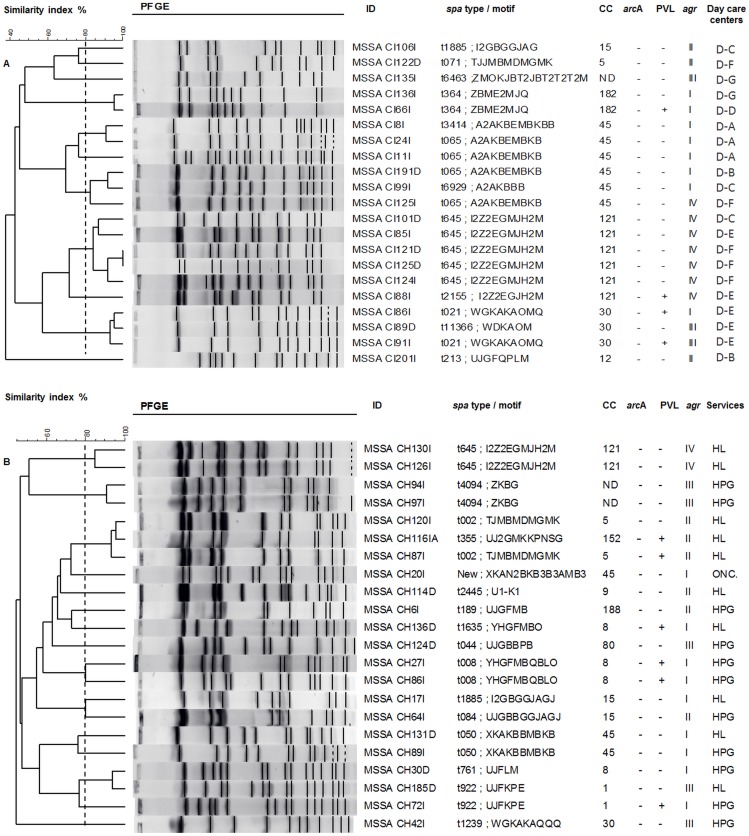
Genetic relatedness among MSSA isolates from (A) day care centers and (B) hospital. UPGMA dendrogram showing genetic relatedness among MSSA isolates as determined by PFGE with *Sma*I. The broken line corresponds to the cut off level (80%) used to define related PFGE clones.

Greater genotypic diversity was detected in MSSA of both populations, as previously described [47], [48]. In this work, MSSA isolates belonged to 15 clonal complexes, the most frequent being CC30 (20.3%), CC45 (17.4%) and CC121 (11.6%). The most common *spa* types were t645 and t021 (both 8.7%), t002 and t050 (both 5.8%) and t1635 (4.3%). PFGE analysis of MSSA isolates, carried out by institution confirmed the genotypic diversity of these isolates (Figs. 3A and B).

### Detection of virulence factor genes and *agr* types

**Figure 4:**
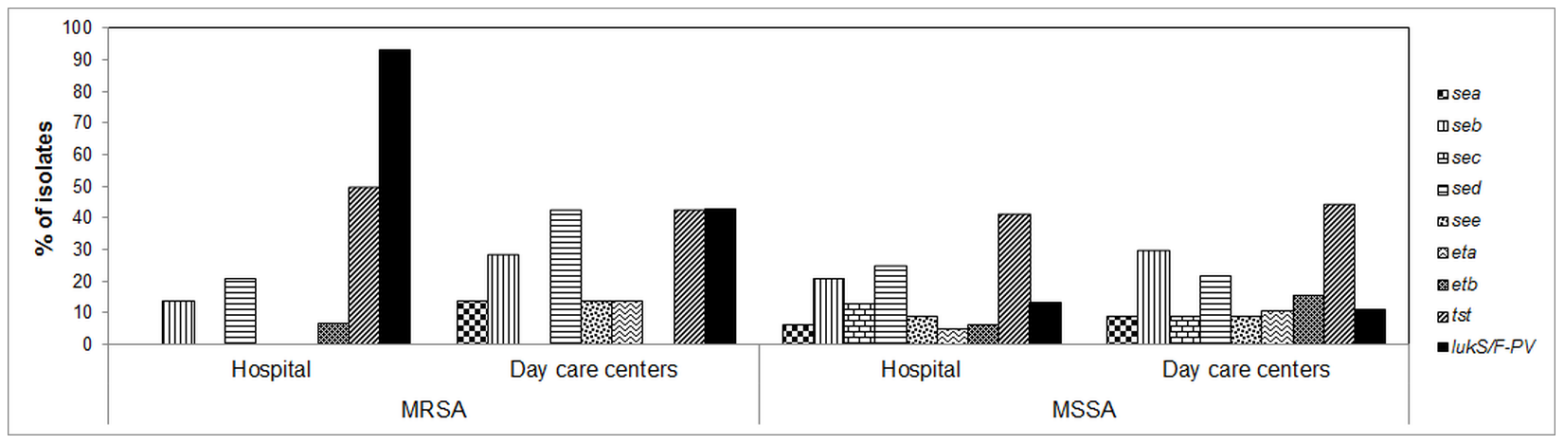
Frequency of distribution of virulence genes among MRSA and MSSA isolates according to institution. Abbreviations: *sea*, *seb*, *sec*, *sed* and *see*: staphylococcal enterotoxin genes A–E; *eta* and *etb*: exfo-liative toxin genes A and B; *tst*: toxic shock syndrome toxin 1 gene; *lukS/F-PVL*: Panton-Valentine Leucocidine.

Detection of virulence factor genes in the 159 *S. aureus* isolates showed that 74.2% (*n* = 118) carry at least one virulence factor gene (Fig. 4). In general, a higher proportion and diversity of virulence factors were detected among MSSA isolates. This finding is of importance because it has been suggested that the presence of virulence factor genes predicts, to a certain extent, the pathogenic capacity of colonizing isolates [23].

Genes *lukS/F-PV* were present in 14.3% (n = 1 0) and 25.8% (n = 23) of DCCs and hospital colonizing isolates, respectively. The frequency for these genes in DCCs is higher than previously reported in a similar study in Cartagena, Colombia (14.3% vs. 5.8%) [26], but it is lower than the one found among children of countries such as China (22.4%) [49]. PVL genes (*lukS/F-PV*) were detected in 76.2% (n = 16) of the MRSA isolates, 42.2% (n = 3) from DCCs and 92.9% (n = 13) from the hospital. The percentage of the PVL genes in hospital MRSA colonizing strains was similar to previously reported for infectious isolates from the child population of the same hospital, with 94.0% of the isolates carrying these genes [50]. The frequency of PVL genes among colonizing MSSA strains was 12.3%, higher than reported among Swiss children (1.6%) [51]. Recent studies have reported on an increase in their frequency in S. aureus (MRSA-MSSA), colonizing and clinical isolates [52]. A hospital MRSA isolate carried the arcA gene for ACME (CC8-SCCmec IVa-t1635), PVL (lukS/F-PV) and agrI. PFGE did not reveal any relationship with the rest of colonizing MRSA isolates or with a few infectious isolates form a previous study and strain USA300-0114 CA-MRSA (ST8-SCC*mec* IVa), included in the PFGE assay as reference strains. Presence of the ACME-*arcA* gene has been correlated with strains USA300 [53], but particularly, most isolates circulates in Colombia are ACME-*arcA* negative, known as the “Latin American variant” clone, USA300-LV [54]. Besides the finding of an ACME-*arcA* colonizing isolate, one infectious strain with this gene was recently reported from Bogotá, the capital city of Colombia [55]. This suggests the importance of a continued surveillance for this type of virulence factors; particularly, it has been suggested that ACME could provide *S. aureus* with an increased survival and colonization ability, conferring a selective advantage and improving its virulence capacity [56].

**Figure 5:**
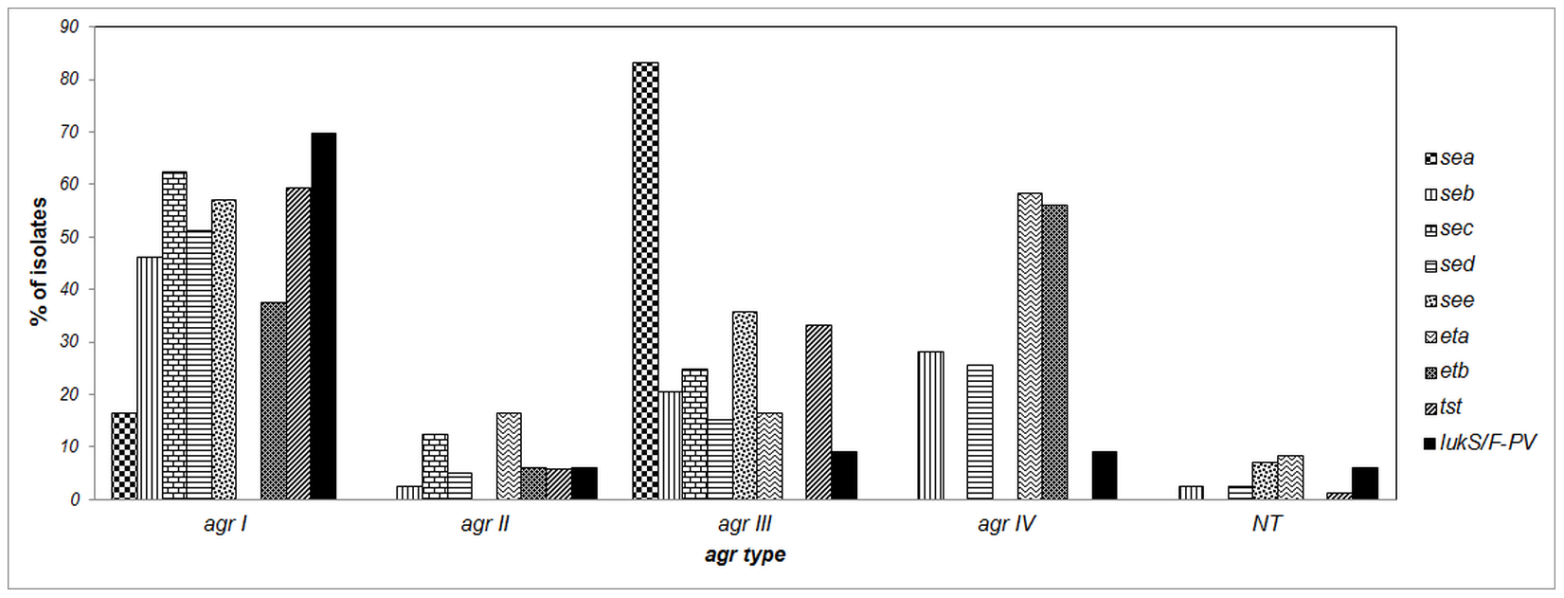
Percentage distribution of virulence genes according to *agr* type in *S. aureus* isolates. Abbreviations: *sea*, *seb*, *sec*, *sed* and *see*: staphylococcal enterotoxin genes A–E; *eta* and *etb*: exfo-liative toxin genes A and B; *tst*: toxic shock syndrome toxin 1 gene; *lukS/F-PVL*: Panton-Valentine Leucocidine genes.

One of the principal regulatory genes of *S. aureus* is *agr*, which controls the temporal expression of most virulence factors [51]. The distribution of virulence factors within each *S. aureus agr* group is shown in Fig. 5. The *agrI* group contained 76.2% of MRSA and 58.0% of the MSSA isolates. Interestingly, these percentages are similar to reports for *agrI* in infectious MRSA isolates, e.g. 71% [57] and up to 96% [58] of. Presumably, there is a correlation between the type of *agr* with the presence of some virulence factor genes or with the genetic background of the strains [59]. Accordingly, in the present study, all the strains that belonged to CC30 were *agr*III, also of 17 CC8 isolates, 15 were *agr*I, and of 10 CC121 isolates, eight were *agr*IV.

In addition, a relationship between virulence factors and clonal complex was detected, thus of 17 CC8 isolates, 16 presented *lukS/F-PV* and 14 *tst* genes. Of 14 CC30 strains, 13 had *tst* and nine, *sea*. Of 10 CC121 isolates, seven carried *eta* and eight *etb*. The importance of this finding resides in that CC121 is known as the “impetigo clone” because of its association with impetigo affected patients, a very contagious condition affecting mostly children, and often related with *eta* and *etb* gene [48]. Most *S. aureus* CC121 isolates were detected in DCCs, results that should guide prevention measures.

Among the limitations of this work are, that as a cross-sectional study it was not possible to detect variations in colonization patterns, e.g. persistent carriers, intermittent carriers or non-carriers. Also, sampling only the nostrils without including other body parts may represent an underestimation of the frequency of MRSA [35], and finally, the inability to detect if colonization originated in the hospital or the community limits our ability to make generalizations about the results.

Nevertheless, our findings provide relevant information on *S. aureus* and MRSA colonization behaviors in the pediatric population at the local level. The finding of colonizing MRSA with similar molecular characteristics to infectious strains demonstrates the importance for public health of monitoring these populations, because of the risks of these strains disseminating and causing infection. Furthermore, the differences in epidemiological characteristics between the populations provide the baseline for the design of control and prevention strategies for colonizing and infectious *S. aureus.*
